# Evaluation of different set-up error corrections on dose–volume metrics in prostate IMRT using CBCT images

**DOI:** 10.1093/jrr/rru033

**Published:** 2014-05-12

**Authors:** Yoshinori Hirose, Mitsuhiro Nakamura, Tsuneyuki Tomita, Kenji Kitsuda, Takuya Notogawa, Katsuhito Miki, Kiyonao Nakamura, Takashi Ishigaki

**Affiliations:** 1 Division of Radiology, Osaka Red Cross Hospital, Osaka, 534-8555, Japan; 2 Department of Radiation Oncology and Image-applied Therapy, Graduate School of Medicine, Kyoto University, 54 Kawahara-cho, Shogoin, Sakyo-ku, Kyoto, 606-8507, Japan; 3 Department of Radiation Oncology, Osaka Red Cross Hospital, Osaka, 534-8555, Japan

**Keywords:** intensity-modulated radiotherapy, set-up error correction, dosimetry, prostate cancer

## Abstract

We investigated the effect of different set-up error corrections on dose–volume metrics in intensity-modulated radiotherapy (IMRT) for prostate cancer under different planning target volume (PTV) margin settings using cone-beam computed tomography (CBCT) images. A total of 30 consecutive patients who underwent IMRT for prostate cancer were retrospectively analysed, and 7–14 CBCT datasets were acquired per patient. Interfractional variations in dose–volume metrics were evaluated under six different set-up error corrections, including tattoo, bony anatomy, and four different target matching groups. Set-up errors were incorporated into planning the isocenter position, and dose distributions were recalculated on CBCT images. These processes were repeated under two different PTV margin settings. In the on-line bony anatomy matching groups, systematic error (∑) was 0.3 mm, 1.4 mm, and 0.3 mm in the left–right, anterior–posterior (AP), and superior–inferior directions, respectively. ∑ in three successive off-line target matchings was finally comparable with that in the on-line bony anatomy matching in the AP direction. Although doses to the rectum and bladder wall were reduced for a small PTV margin, averaged reductions in the volume receiving 100% of the prescription dose from planning were within 2.5% under all PTV margin settings for all correction groups, with the exception of the tattoo set-up error correction only (≥5.0%). Analysis of variance showed no significant difference between on-line bony anatomy matching and target matching. While variations between the planned and delivered doses were smallest when target matching was applied, the use of bony anatomy matching still ensured the planned doses.

## INTRODUCTION

Intensity-modulated radiotherapy (IMRT) techniques allow the safe delivery of high-dose radiation to the prostate while sparing adjacent normal structures, including the rectum and bladder [1]. This requires accurate daily targeting throughout the entire course of IMRT. A number of researchers have investigated the localization and quantification of prostate displacement using implanted fiducial markers and several modalities, including kV X-ray planar images and cone-beam computed tomography (CBCT) [2–7]. Hammond *et al*. noted that residual set-up errors in the prostate with respect to the planned position remain, even after bone alignment [7]. In addition, several papers have described appropriate set-up error corrections for reducing residual set-up error using repeated CT scans [8, 9]. Hoogeman *et al*. and Snir *et al*. reported that residual set-up error in the prostate could be reduced using the data of four to five repeated CT scans with off-line corrections [8, 9]. Meanwhile, some researchers have analysed dose–volume metrics of the prostate, rectum and bladder after bone matching or target matching (using CT or CBCT images to assess the accuracy of the initial treatment plan) [5, 10]. To date, however, few studies have comprehensively investigated dose–volume metrics under the presence of residual set-up errors and different planning target volume (PTV) margin settings.

Here, we investigated the effect of different set-up error corrections on dose–volume metrics in IMRT for prostate cancer under different PTV margin settings using CBCT images.

## MATERIALS AND METHODS

### Patients

The study enrolled 30 consecutive patients who underwent IMRT for localized prostate cancer at our institution between April 2011 and November 2011 (Table 1). Written informed consent for IMRT was obtained from each patient before treatment planning. Patients were provided with written and verbal instructions regarding bowel and bladder preparation before simulation and treatment. They took one capsule of magnesium oxide (330 mg) orally three times per day to encourage defecation, and were instructed to empty their bowel and bladder, and then drink 300–500 ml water 1 h before the CT simulation and before each treatment.

**Table 1.** Patient characteristics

Age (years)		
	Median (range)	71 (61–81)
Prescription dose		
	70 Gy	8
	74 Gy	12
	78 Gy	10
CTV		
	Prostate + 1/3 SV	9
	Prostate + 2/3 SV	20
	Prostate + SV	1

CTV = clinical target volume, SV = seminal vesicles.

### Planning CT data acquisition and IMRT planning

**Fig. 1.**
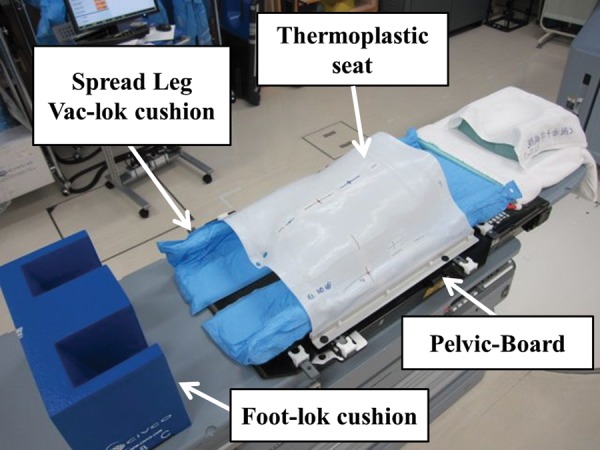
The Hip-fix system, which includes the Pelvic-Board, Spread Leg Vac-lok cushion, thermoplastic seat, and Foot-lok cushion.

At the CT simulation, patients were positioned supine on the couch with the Hip-fix system, which includes the Pelvic-Board, Spread Leg Vac-lok cushion (CIVCO Medical Solutions, Kalona, IA), thermoplastic seat, and Foot-lok cushion (Med-Tech, Orange City, IA, USA) (Fig. 1). The Hip-fix system was developed in order to facilitate maintaining natural width of leg opening and leg rotations [11]. Planning CT images were then acquired using a 4-slice CT simulator (Light speed plus; General Electric Medical Systems, Waukesha, WI) with a 2.5-mm slice thickness without a gap from the iliac crest to 80 mm below the ischial tuberosities. When large amounts of bowel gas and stool in the rectal vault were observed, the patients were asked to empty their bowels and bladder by radiological technicians. After a second bowel and bladder preparation, a planning CT scan was done on the same day. A single experienced radiation oncologist removed bowel gas with a tube when large amounts of bowel gas were still present.

After transfer of the CT images to a treatment-planning system [Eclipse Helios, ver. 8.6.15 (Varian Medical Systems, Palo Alto, CA)], the prostate, seminal vesicles (SVs), outer rectal wall, outer bladder wall, small bowel, and large bowel were manually contoured by the same medical physicist to eliminate interobserver variation. The rectal wall was automatically generated from the outer rectal wall using a wall-extraction function with a wall thickness of 4 mm from 10 mm below the apex of the prostate to 10 mm above the tips of the SVs. The bladder wall was also generated from the outer bladder wall in the same manner as the rectal wall, with a wall thickness of 4 mm. Clinical target volume (CTV) was determined as follows: (i) the union of prostate with the proximal one-third of the SVs for nine patients with a low- or intermediate-risk prostate cancer in Stage B, (ii) the union of prostate with the proximal two-thirds of the SVs for 20 patients with high-risk prostate cancer in Stage B, and (iii) the union of the prostate with the SVs for one patient with Stage T3b prostate cancer. In addition, the following two PTV margin settings were employed in the present study: (i) 8-mm margins isotropically, except for a 5-mm margin posteriorly (in the direction towards the rectum) and superiorly (in the caudal direction) (PTV_8/5_ groups), and (ii) 5-mm margins isotropically (PTV_5/5_ groups). Finally, all contours were reviewed by the radiation oncologist.

An IMRT plan was designed for each of the above PTV settings using Eclipse. The prescription doses were 70 Gy for eight patients, 74 Gy for 12 patients, and 78 Gy for 10 patients, at 2 Gy per fraction. Seven coplanar ports with gantry angles of 50°, 95°, 150°, 180°, 210°, 265° and 310° were selected for dose calculation. Beam energy and dose rate were a 10-MV photon beam and 300 MU/min, respectively. The well-commissioned Analytical Anisotropic Algorithm (ver. 8.6.15) with heterogeneity correction was used for dose calculation. Details regarding the dose–volume constraints were as follows (Table 2).

**Table 2.** Dose–volume constraints in IMRT planning

Structure	Dose–volume constraints
PTV	Maximum dose ≤110%
	V_90%_ > 96% (98%)
	D_95%_ > 90% (95%)
	99% ≤ Mean dose ≤103%
Rectum wall	V_40 Gy_ ≤ 60%
	V_60 Gy_ ≤ 30%
	V_70 Gy_ ≤ 20%
	V_78 Gy_ < 1%
Bladder wall	V_40 Gy_ ≤ 60%
	V_70 Gy_ ≤ 35%
Large bowel	V_65 Gy_ ≤ 0.5 ml
Small bowel	V_60 Gy_ ≤ 0.5 ml

Values in parentheses are preferable. PTV = Planning target volume, V_90%_ = volume receiving 90% of the prescription dose, D_95%_ = dose received by 95% volume, V_xx Gy_ = the volume receiving more than xx Gy.

#### PTV

(i) The maximum dose should be <110%; (ii) the volume receiving 90% of the prescription dose should generally be≥ 96% (≥98% is preferable); (iii) the dose received by 95% volume should generally be ≥90% (≥95% is preferable); and (iv) the mean dose should generally be 99–103% of the prescription dose.

#### Rectum wall

(i) No more than 60% of the rectum wall volume should receive >40 Gy; (ii) no more than 30% of the rectum wall volume should receive >60 Gy, (3) no more than 20% of the rectum wall volume should receive >70 Gy, and (4) no more than 1% of the rectum wall volume should receive >78 Gy.

#### Bladder wall

(i) No more than 60% of the bladder wall volume should receive >40 Gy, and (ii) no more than 35% of the bladder wall volume should receive >70 Gy.

#### Large bowel

No more than 0.5 ml of the large bowel should receive >65 Gy.

#### Small bowel

No more than 0.5 ml of the small bowel should receive >60 Gy.

### Orthogonal kV X-ray planar images and CBCT data acquisition

Prior to irradiation, patients were first aligned based on tattoos on the skin indicating the planning isocenter (IC) location with a system of wall-mounted alignment lasers. A pair of orthogonal kV X-ray planar images was then obtained using the on-board imager systems of a Clinac iX (Varian Medical Systems, Palo Alto, CA). These kV X-ray planar images were manually aligned to their corresponding digitally reconstructed radiographs by therapists and independently verified by physicians. The manual alignment provided left–right (LR), anterior–posterior (AP), and superior–inferior (SI) couch shifts, which were applied to the treatment couch. Respective couch shift values show the matching difference between the laser and kV image.

After correcting initial set-up errors based on bony anatomy, a CBCT scan was sequentially acquired on first, second and third fractions, and thereafter on the first day of the week. The maximum reconstructed field-of-view was a circle of 450 mm diameter and 180 mm in length. For each patient, seven to 14 (average nine) CBCT datasets were acquired during the whole treatment course of 7–8 weeks. When target positions were deviated from the PTV, we confirmed the target position on second CBCT images after the initial target matching.

The prostate, bladder wall and rectum wall were again determined (in the same manner as described in ‘Planning CT data acquisition and IMRT planning’) on the acquired CBCT datasets. Center of mass (COM) mismatches for the prostate between the planning CT and each CBCT dataset were then calculated in the LR, AP and SI directions. In the present study, rotational errors were not evaluated. Subsequently, the systematic variation ∑ (standard deviation of the average shifts for the patient cohort) and the random variation σ (root-mean-square of the standard deviations of the shifts for all patients) were calculated for the LR, AP and SI directions.

### Set-up corrections

To evaluate interfractional variations in dose–volume metrics under different set-up error corrections, six set-up error corrections were employed as follows.

#### SE_tattoo_

Patients were aligned only based on the tattoos.

#### SE_bone_

After aligning patients based on the tattoos, set-up error was corrected on-line based on bony anatomy using orthogonal kV X-ray planer images.

#### SE_COM1_

Set-up error on second and later fractions was corrected off-line based on the COM of the first CBCT acquisition only.

#### SE_COM2_

Set-up error on the third and later fractions was corrected off-line based on the averaged COM of the first two CBCT acquisitions.

#### SE_COM3_

Set-up error on the fourth and later fractions was corrected off-line based on the averaged COM of the first three CBCT acquisitions.

#### SE_COMall_

Set-up error was corrected virtually on-line based on the COM of every CBCT acquisition.

### Dose recalculation on CBCT images under the different set-up corrections above

After incorporating these set-up errors into planning the IC position, dose distributions were recalculated on CBCT images under the same conditions as in planning using the electron density conversion table obtained from the planning CT scanner. Although CBCT-based treatment plans are dosimetrically comparable with planning CT-based plans [12], there are some differences in the electron density conversion tables between planning CT and CBCT [13]. In the present study, after comparing the IC dose on CBCT images with that on the planning CT, the IC dose on CBCT images was normalized to that on the planning CT plan based on the results presented by Hatton *et al.* [10]. Accordingly, monitor units were different from the planned ones.

Dose–volume metrics of the prostate, rectum wall and bladder wall were calculated from the relevant histograms. As for the rectum wall and bladder wall, V_90%_ and V_100%_ (volumes receiving 90% and 100%, respectively, of the prescription dose) were obtained, because higher doses have more impact on the complication probability [14]. Results for different set-up corrections were compared using analysis of variance (ANOVA) with Dunnett tests, where *P* < 0.05 was considered statistically significant.

## RESULTS

### COM distance

**Fig. 2.**
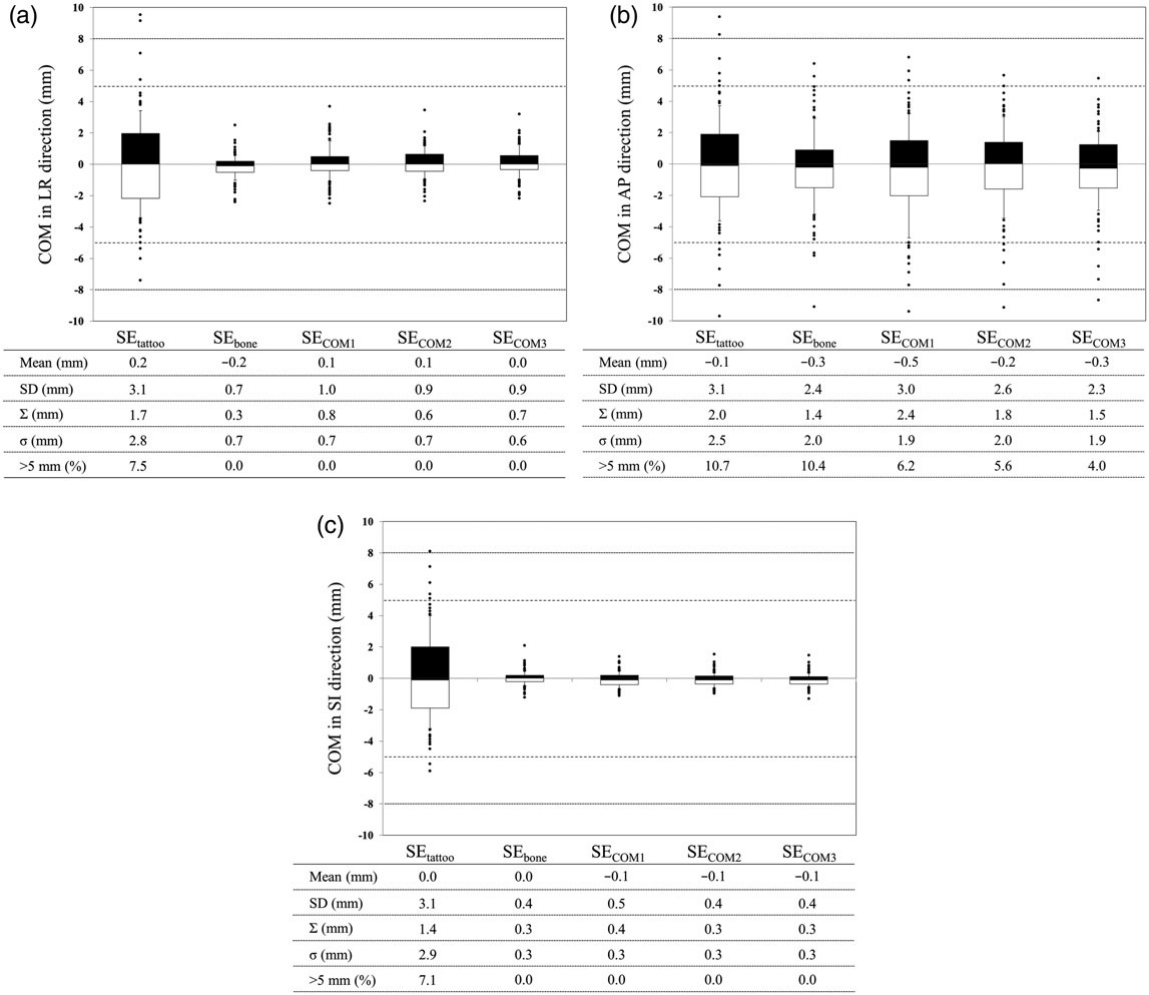
COM distance in the (**a**) LR, (**b**) AP, and (**c**) SI direction. Dose analysis parameters are graphically presented in box plots (showing medians, and 25th and 75th percentiles) with whiskers (10th and 90th percentiles); outliers are shown as dots. ‘ >*n* mm (%)’ means a frequency of displacement of >*n* mm.

The COM distances with the respective set-up error corrections are shown in Fig. 2. Mean ± SD of set-up error in the LR, AP and SI directions was 0.2 ± 3.1 mm, −0.1 ± 3.1 mm, and 0.0 ± 3.1 mm, respectively, in the SE_tattoo_ groups, versus −0.2 ± 0.7 mm, −0.3 ± 2.4 mm, and 0.0 ± 0.4 mm, respectively, in the SE_bone_ groups, showing a significant difference between these two groups (*P* < 0.05). Mean ± SD of set-up errors for the SE_COM_ groups (excluding the SE_COM1_ groups) was comparable with that of the SE_bone_ groups in all directions (Fig. 2).

∑ and σ were <1.0 mm in the LR and SI directions, respectively, except in the SE_tattoo_ groups. ∑ was < 2.0 mm in the AP direction, except in the SE_COM1_ groups (2.4 mm)_,_ in which σ was <2.0 mm. ∑ in the SE_COM3_ groups was comparable with that in the SE_bone_ groups in the AP direction. The frequency of >5.0 mm displacement in the AP direction was 10.7% in the SE_tattoo_, 10.4% in the SE_bone_, 6.2% in the SE_COM1_, 5.6% in the SE_COM2_, and 4.0% in the SE_COM3_ groups. Meanwhile, the frequency of > 8.0 mm displacement in the AP direction was 2.2% in the SE_tattoo_, 1.3% in the SE_bone_, 0.5% in the SE_COM1_, 0.6% in the SE_COM2_, and 0.7% in the SE_COM3_ groups.

### Dosimetric evaluation

**Fig. 3.**
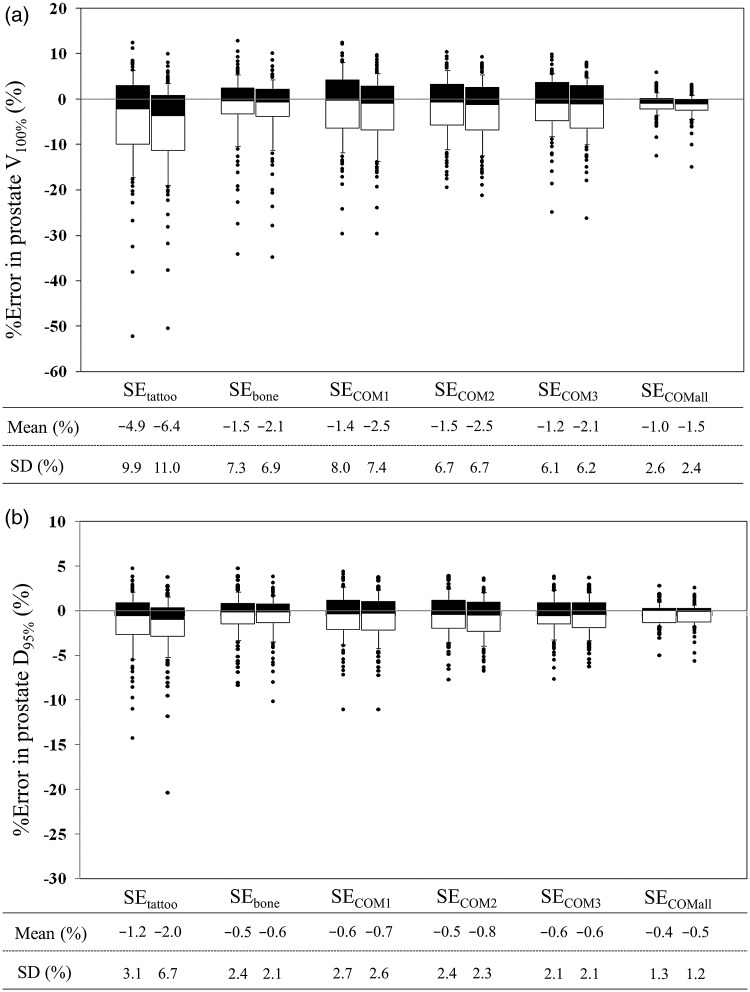
Interfractional variation in prostate (**a**) V_100%_ and (**b**) D_95%_. Data are shown in percentage points. In each set-up correction policy, the left and right box plots show data in the PTV_8/5_ and PTV_5/5_ groups, respectively. Dose analysis parameters are graphically presented in box plots (showing medians, and 25th and 75th percentiles) with whiskers (10th and 90th percentiles); outliers are shown as dots.

Figure 3 shows interfractional variation in prostate V_100%_ and D_95%_ (the dose delivered to 95% of the prostate volume) for the PTV_8/5_ and PTV_5/5_ groups. Averaged reductions in prostate V_100%_ and D_95%_ were within 2.5% and 1.0%, except for the SE_tattoo_ groups. ANOVA showed a statistically significant difference between the SE_tattoo_ and SE_bone_ groups (*P* < 0.05); however, no significant difference was seen between the SE_bone_ and other SE_COM_ groups.

**Fig. 4.**
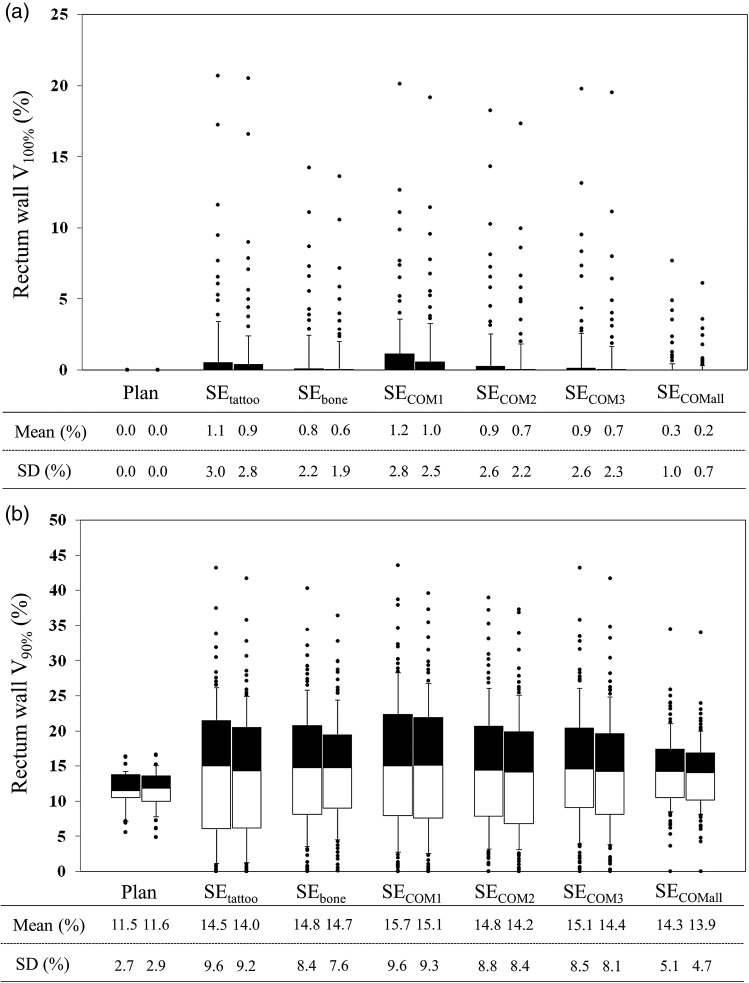
Interfractional change in rectum wall (**a**) V_100%_ and (**b**) V_90%_. For each set-up correction policy, the left and right box plots show data in the PTV_8/5_ and PTV_5/5_ groups, respectively. Dose analysis parameters are graphically presented in box plots (showing medians, and 25th and 75th percentiles) with whiskers (10th and 90th percentiles); outliers are shown as dots.

**Fig. 5.**
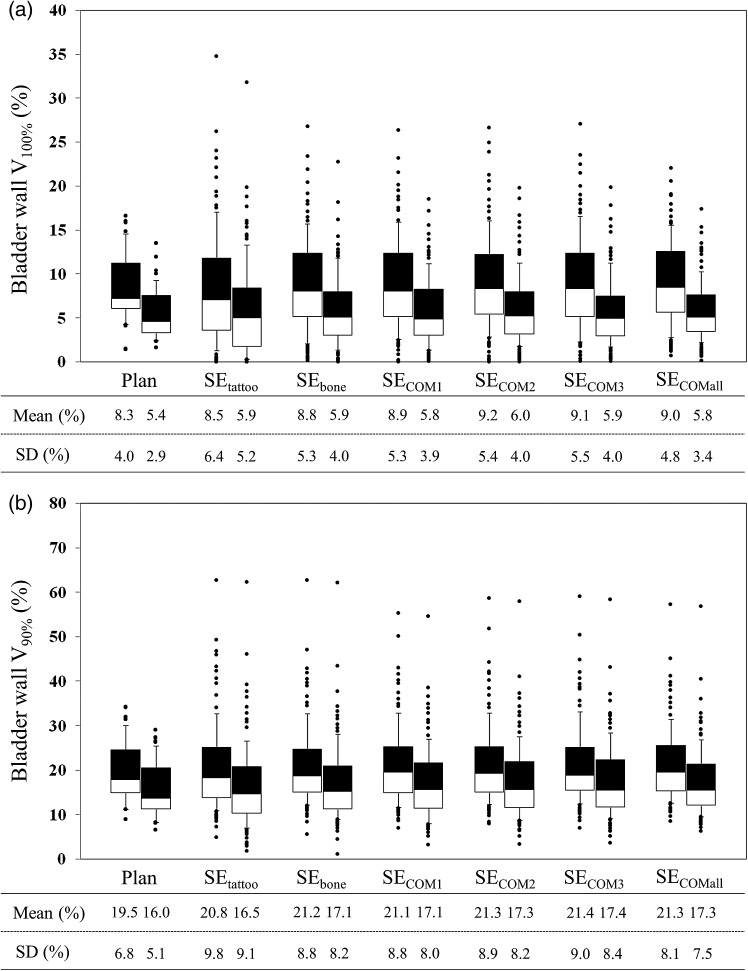
Interfractional change in bladder wall (**a**) V_100%_ and (**b**) V_90%_. For each set-up correction policy, the left and right box plots show data in the PTV_8/5_ and PTV_5/5_ groups, respectively. Dose analysis parameters are graphically presented in box plots (showing medians, and 25th and 75th percentiles) with whiskers (10th and 90th percentiles); outliers are shown as dots.

Compared with the planned dose for the rectum wall and bladder wall, V_100%_ and V_90%_ were delivered an excessive dose (Figs 4 and 5). Significant differences in these dose–volume metrics were seen between the SE_tattoo_ and other groups (*P* < 0.05).

Compared with the PTV_8/5_ margin groups, the SD of the prostate V_100%_ and D_95%_ were increased, while the rectum V_100%_ and V_90%_ and bladder V_100%_ and V_90%_ were decreased for the PTV_5/5_ margin groups (Figs 4 and 5); however, there were no significant differences in averaged dose–volume metrics between the PTV_8/5_ margin and PTV_5/5_ margin groups.

## DISCUSSION

Hammond *et al*. evaluated residual set-up errors using CBCT images, and reported that the total mean ± SD for five patients was 0.7 ± 0.6 mm, 1.6 ± 1.2 mm and 1.4 ± 1.0 mm in the LR, AP and SI directions, respectively [7]. Compared with the results of their study, residual set-up errors based on bony anatomy were smaller in our study. One possible reason for this difference is a decrease in statistical uncertainty, given the small number of patients (*n* = 5) in their study. Recently, many studies have reported on set-up error corrections using implanted fiducial markers. Interestingly, some of these reported the presence of prostate motion against the bony anatomy [15–17]. McNair *et al.* reported that systematic error (calculated using gold markers rather than bony anatomy) was >2.0 mm different in at least one direction in 50% of patients (with a difference >5.0 mm in 13% of patients) using fiducial markers as opposed to bony anatomy [17]. In the present study, fiducial markers were not implanted for all participating patients, and the COM mismatches were calculated based on the delineated prostate. Nevertheless, our results for prostate displacement from the bony anatomy were comparable with their results. Displacement in the AP direction may be attributable to contraction of pelvic muscles or a change in rectal volume [18, 19]. Of all set-up error correction policies, the SE_COM1_ groups had the largest ∑ in the AP direction (2.4 mm), indicating that systematic set-up error cannot be reduced by a single measurement only. Prostate position in the first fraction is less representative; a decrease in systematic set-up errors therefore requires multiple positional data for the prostate.

In order to reduce the risk of toxicity, it is important to set valid dose–volume constraints. According to the report published by Michalski *et al*., most dose–volume parameters significantly associated with late rectal toxicity considered doses ≥60 Gy [14]. Our dose–volume constraints to the rectum wall (Table 2) satisfied their recommended dose–volume limits [14]. However, it is generally known that interfractional variations in doses to organs at risk (OARs) occur. Kupelian *et al*. [5], Hatton *et al*. [10] and van Haaren *et al.* [20] all reported that rectal doses were generally higher than the planned dose. These results are consistent with those of our present study (Figs 4 and 5). Variations in the volume and shape of OARs (and a different set-up from the planning) cause interfractional variations in doses to OARs, which would result in unintentional toxicities, even with well-established dose–volume constraints; therefore, the preparation prior to treatment and the establishment of a matching protocol are required to control the variations. Figures 4 and 5 revealed that set-up error correction based on target matching was effective in minimizing the interfractional variations in doses to OARs, while the tattoos-based set-up error correction caused the largest ones. These results indicated that it is preferable to employ target matching when bowel gas and stool are present.

Our results showed that the margin reduction plan allowed sparing of the bladder and rectal high-dose regions as indicated by Hammond *et al.* [7]. Our present retrospective analysis found that no significant differences in the prostate V_100%_ and D_95%_ between the PTV_8/5_ margin and PTV_5/5_ margin groups were observed, even under bony set-up correction when employing our CTV determination policy and immobilization system. Recently, Engels *et al.* have reported a potential danger posed by image-guidance techniques with regard to PTV margin reduction [21]; thus, the differences in a treatment protocol, including CTV determinations, PTV margin settings and immobilization systems, would influence treatment outcomes, even with full use of the image-guidance function.

Three limitations of our study warrant mention. (i) CBCT was not scanned at the time of delivery of every fraction. Certainly, it is preferable to acquire CBCT data for accurate evaluation because the shape of the OAR and prostate changes daily; however, we decided not to do this with a view to minimizing exposure [22]. From our results, the number of CBCT data was sufficient to evaluate dosimetry. (ii) Intrafractional prostate motion was not considered. Several reports on the intrafractional motion have been published to date [23–25]. The representative intrafractional motions are respiratory motion and baseline drift of prostate position. In general, respiratory motion of the prostate was mostly less than our PTV margin size of 5 mm [23, 24]; therefore, respiratory motion would have little influence on the dosimetry. However, baseline drift will cause unpredictable systematic errors [25], which cannot be quantified on CBCT images. To address this issue, real-time monitoring is required. (iii) Deformable image registration (DIR) methods were not used. Song *et al*. previously reported that a change in the volume and shape of organs had only a moderate influence on dosimetry for prostate cancer, even if DIR methods were used [26]; accordingly, we expected that no significant difference would be seen in dosimetry between delivery with and without DIR.

## CONCLUSION

We retrospectively analyzed the dose delivered to the prostate, rectal wall and bladder wall in a total of 270 CBCT sets from 30 consecutive prostate cancer patients treated with IMRT under the six different set-up error corrections and two PTV margin settings. The dose distributions obtained with a set-up correction based on tattoo only were significantly worse than those yielded by the others. As expected, variations between the planned and delivered doses were smallest when target matching was applied. However, the use of bony set-up correction still ensured the delivery of planned doses in IMRT for prostate cancer. In addition, doses to OARs were reduced by shrinking the PTV margin size; however, interfractional variations in dose–volume metrics were almost fully consistent in the PTV_8/5_ and PTV_5/5_ groups, even under different set-up error corrections.

## FUNDING

This work was supported by a Grant-in-Aid for Young Scientists (B) from the Ministry of Education, Culture, Sports, Science, and Technology, Japan [Grant number 23791408]. Funding to pay the Open Access publication charges for this article was provided by a Grant-in-Aid for Scientific Research from the Japanese College of Medical Physics for 2012.
